# Biomechanical Assessment of Post-Stroke Patients’ Upper Limb before and after Rehabilitation Therapy Based on FES and VR

**DOI:** 10.3390/s22072693

**Published:** 2022-03-31

**Authors:** Daniela Montoya, Patricio Barria, Carlos A. Cifuentes, Luis F. Aycardi, André Morís, Rolando Aguilar, José M. Azorín, Marcela Múnera

**Affiliations:** 1 Biomedical Engineering Department, Colombian School of Engineering Julio Garavito, Bogota 111166, Colombia; daniela.montoya@mail.escuelaing.edu.co (D.M.); luis.aycardi-c@mail.escuelaing.edu.co (L.F.A.); marcela.munera@escuelaing.edu.co (M.M.); 2 Club de Leones Cruz del Sur Rehabilitation Center, Punta Arenas 6210133, Chile; pbarria@rehabilitamos.org (P.B.); laboratorios@rehabilitamos.org (A.M.); 3 Department of Electrical Engineering, University of Magallanes, Punta Arenas 6210427, Chile; rolando.aguilar@umag.cl; 4 Brain-Machine Interface Systems Lab, Systems Engineering and Automation Department, Miguel Hernández University of Elche UMH, 03202 Elche, Spain; jm.azorin@umh.es; 5 School of Engineering, Science and Technology, Universidad del Rosario, Bogota 111711, Colombia

**Keywords:** exergaming, functional electrical stimulation (FES), kinematics, rehabilitation, stroke, virtual reality (VR)

## Abstract

Stroke is a medical condition characterized by the rapid loss of focal brain function. Post-stroke patients attend rehabilitation training to prevent the degeneration of physical function and improve upper limb movements and functional status after stroke. Promising rehabilitation therapies include functional electrical stimulation (FES), exergaming, and virtual reality (VR). This work presents a biomechanical assessment of 13 post-stroke patients with hemiparesis before and after rehabilitation therapy for two months with these three methods. Patients performed two tests (Maximum Forward Reach and Apley Scratching) where maximum angles, range of motion, angular velocities, and execution times were measured. A Wilcoxon test was performed (*p* = 0.05) to compare the variables before and after the therapy for paretic and non-paretic limbs. Significant differences were found in range of motion in flexion–extension, adduction–abduction, and internal–external rotation of the shoulder. Increases were found in flexion–extension, 17.98%, and internal–external rotation, 18.12%, after therapy in the Maximum Forward Reach Test. For shoulder adduction–abduction, the increase found was 20.23% in the Apley Scratching Test, supporting the benefits of rehabilitation therapy that combines FES, exergaming, and VR in the literature.

## 1. Introduction

Stroke is a syndrome in which focal loss of brain function is rapidly generated without any apparent cause of a vascular origin. This syndrome varies seriously from recovery in one day to incomplete recovery, severe disability, and death [1]. Stroke is considered one of the most devastating neurological conditions. Globally, 5.5 million people die from a cerebrovascular accident (CVA) per year, and 44 million survivors end up with a disability [2]. Moreover, almost one in four men and one in five 45-year-old women can suffer a CVA if they live up to 85 years old. The overall incidence rate is around 2 to 25,000 inhabitants [3]. In 2023, there is an expected increase of patients experiencing a CVA of about 30% [3]. In the United States, CVA is the third cause of death and the first disability rate. Approximately 550,000 people suffer from a CVA per year, 150,000 dies, and survivors have various degrees of neurological deterioration [4].

CVA affects a person physically, emotionally, and socially, and it is known that even if the mortality rates for a stroke decrease, the likelihood that the person has complications in activities of daily living (ADLs) is increasing [5,6]. More than 80% of people who survive a stroke have hemiparesis. Of them, it is estimated that 70% have residual deterioration [5,6]. Furthermore, post-stroke effects generate different musculoskeletal complications, such as spasticity, dystonia, muscle contracture, loss of force and skill, decreased range of joint movement, lack of speed, accuracy, and coordination [6,7]. In addition, 18% of them have severe hemiparesis [7]. Spasticity after a stroke occurs in 30% of patients [8]. In addition, dystonia or movement disorders after a stroke accident account for 1–4% [9]. Between 55% and 85% of stroke survivors have a paretic arm that leads to motor limitation and difficulties in daily life activities. Such condition generates weakness, spasticity, and unwanted muscle synergies [10]. After the acute phase, 20% recover full arm function six months after stroke. Therefore, early and intensive intervention is essential to promote motor recovery of the paretic arm [6,10].

### 1.1. Motivation

Rehabilitation in post-stroke patients is based on the training of patients with compensatory strategies [11]. On the one hand, according to several studies, motivation critically influences motor and functional outcomes for people with orthopedic and neurological disorders [12]. A high adherence rehabilitation program is indicative of motivation [12,13,14,15]. The lack of motivation is a barrier to physical activity and training after stroke [12,16,17]. Various strategies are used to motivate stroke patients in clinical practice with positive outcomes such as virtual reality [12].

On the other hand, post-stroke patients can experience pain, weakness, sensory loss, impaired dexterity, and lack of coordination in upper limbs. For this reason, upper limb motion has been a fundamental goal in rehabilitation [5]. Currently, stroke rehabilitation focuses on training patients with countervailing strategies. In other words, doctors encourage patients to develop greater strength, speed, resistance, and accuracy of the affected joints’ movements, generate increased independence, and improve their development in daily life activities [11].

Post-stroke patients should maintain rehabilitation training to prevent degeneration of physical function and prevent other related complications from arising. New rehabilitation approaches have shown promising results in the area. Dobkins [11] studied three rehabilitation methods: virtual reality (VR), exergaming, and electrical neurostimulation.

VR presents sensory feedback as subjects undergo a virtual environment to witness their own body in motion. Virtual reality aims to reduce kinetosis and the time delay between the visual information received by the subject and the movements performed. In addition, there is an expected increase in the subject’s motivation, since the training has a more ludic aspect [18,19]. VR is a relatively recent approach and aims to allow the simulated practice of functional tasks with a much higher frequency than traditional therapies [19,20,21]. Some studies review the evidence for VR therapy in an adult post-stroke population in virtual environments and on commercially available gaming systems [22]. In addition, Mekbib et al. [23] evaluated the therapeutic potential of VR compared to occupational therapies in post-stroke patients. The authors demonstrated that VR is a promising therapeutic technology in the rehabilitation of post-stroke patients.

The second approach, “exergaming”, is defined by Pirovano et al. [24] as an exercise with a game integrated into its structure. Another definition is provided by Barry et al. [25]. For them, the term “exergaming” refers to computer games that promote physical movements. As for its operation, real-time motion detection is combined with video games that can help motivate people to perform the exercises correctly. Both virtual reality and “exergaming” aim to have the maximum voluntary activity of the patient, which are precise repetitions of the task, frequency, intensity, changes of virtual environments, and gradual increases to have greater complexity of the assigned task [26,27]. Previous studies have reported significant improvements in functional outcomes when using “exergaming” as a complementary therapy in stroke rehabilitation [28]. Yazuver et al. [29] concluded that “exergaming” as a conventional stroke rehabilitation program has more significant potential to improve motor function in the upper limb in post-stroke patients. Furthermore, the “exergaming” procedure, compared to conventional exercises, does not require the intervention of a physiotherapist and has potential for telerehabilitation [26]. It also generates a greater quantity and quality of upper limb movements. Therefore, it promotes the connectivity of the motor system, improving the functional state after a stroke [28].

Finally, functional electrical stimulation (FES) uses assistive electrical stimulation devices to activate muscles precisely and intensively to perform functional tasks directly [30]. FES is a commonly used modality in physical therapy. Among them, FES is a promising technology for assisting upper extremity motor functions in post-stroke rehabilitation [31]. FES is applied so that the electric current can contract the muscle with the necessary force and the right moment to fulfill the following functions in general: grasping, releasing, standing, and walking, among others. Functional electrical stimulation aims to improve strength, reduce pain, and reduce subluxation in the upper extremity and the shoulder. FES is used on the supraspinatus and posterior deltoid muscles. On the other hand, the stimulation aims to improve wrist and finger extension [32]. According to Lane et al. [33], kinematic evidence has been provided that FES application in the upper limb (UL) and the interscapular muscles of stroke patients with motor impairment of the UL reduces trunk tilt and increases shoulder flexion and elbow extension.

### 1.2. Related Works

Several studies have explored the movement and rehabilitation of the upper limb for stroke patients based on the strategies mentioned above and constitute the development background here presented. Molina et al. [34] analyzed the joint movement of the elbow, shoulder, and trunk in post-stroke patients. Based on their joint RoM, the authors concluded that the movements of the trunk and elbow in the sagittal plane decrease in the drinking activity of a vessel, and shoulder and trunk movements in the coronal plane increased [32]. Hingtgen et al. [35] assessed upper limb movement in post-stroke patients with hemiparesis. The authors created a kinematic model based on the time of movement, range of movement, maximum angular speed, percentage of reach where the maximum speed occurs. They concluded that the unaffected arm showed a greater range of motion and angular velocity than the affected arm [35]. Cuesta-Gómez et al. [36] centered on the joint movement analysis of the thorax, shoulder, and elbow in the sagittal plane during the reaching activity. Using FES, they observed that there is an increase in shoulder flexion and elbow extension. Hughes et al. [37] examined the total movement time, maximum velocity, and spectral spectrum in post-stroke patients (in the ARAT block, drink, and pour water tasks) and found a very high correlation and concordance. Finally, the importance of movement analysis measures for post-stroke patients was highlighted by Lane et al. [33] as objective information on the performance and progress of therapy.

This work aims to use a motion caption system to assess the kinematics of the upper limb in patient post-stroke patients before and after a rehabilitation process with virtual reality (VR), exergaming, and functional electrical stimulation (FES) developed over a two months.

## 2. Materials and Methods

To evaluate the effectiveness of virtual reality therapy, exergaming and FES in post-stroke patients with a paretic limb, the non-paretic and paretic extremities were compared before and after eight weeks of therapy in terms of their joint motion. Movement analysis was performed using the VICON System (Oxford Metrics, Oxford, UK and Polygon software) while patients performed two tests: (i) the Maximum Forward Reach Test and (ii) the Apley Scratching Test.

### 2.1. Participants

This study was carried out with 13 post-stroke patients (4 women and 9 men). Patients were between 40 and 70 (56.61 ± 14.16) years old, weighed between 63 and 85 (74.76 ± 10.48) kg, and were between 1.60 and 1.75 (1.69 ± 0.052) m tall. All patients had hemiparesis: three patients on the right side, and ten patients on the left side. The duration from the time of stroke to the start of therapy was between (2.08 ± 1.28) years with a range of 1–4 years. Fugl–Meyer Assessment (FMA) was implemented. Table 1 presents the demographic and clinical characteristics of the patients, who are established under medical control and pharmacological treatment as appropriate.

Inclusion criteria were: hemorrhagic or ischemic stroke, a minimum of six months after acute infarction/onset of the disease, partial upper extremity motor function, unilateral upper extremity paresis, full passive range of motion in upper extremities, or at least achieve neutral position and altered muscle tone maximum 2 of the modified Ashworth scale in elbow flexion and extension.

Patients are excluded if they have peripheral nervous system pathology, epilepsy, no cognitive ability to follow the study instructions, musculotendinous shortening, pregnancy, amaurosis, visual disturbances limiting interaction with serious video games, use of implanted devices, unstable joints of the upper limbs or fixed contracture, upper extremity pain of musculoskeletal origin, patients with the normal functioning of upper extremity motor function, and patients with complete paralysis of the affected upper limb.

The Ethics Committee of the Club de Leones Cruz del Sur Rehabilitation Center (Chile) approved the intervention, and all the participants signed informed consent. At the beginning of each trial, the researchers explained each volunteer’s experimental setup and device’s functionality.

### 2.2. Rehabilitation

**Figure 1:**
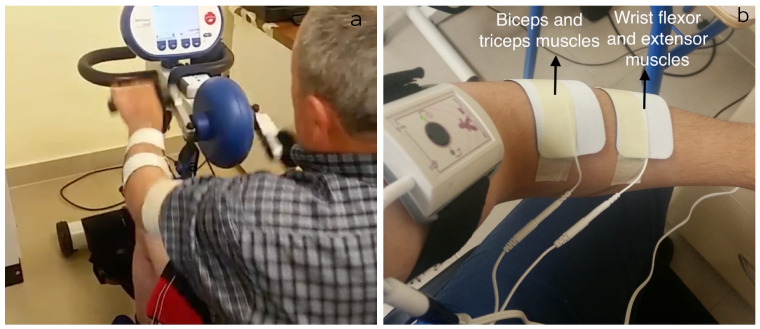
(**a**) Motorized upper limb cycle ergometer (MOTOMED Viva2 REck, Reck, Baden-Wurttemberg, Germany), and (**b**) Placement of electrodes on the bicep, tricep, wrist flexion and extension muscles.

The virtual-assisted rehabilitation consisted of 16 therapy sessions performed two days per week with 60 min per session. At each session, the subject conducted two activities. The first activity lasted 30 min and consisted of a multi-channel FES in the paretic upper limb, which was synchronized with an IMU sensor. The second activity was “exergaming” with a VR headset and lasted another 30 min. The FES therapy was performed through active exercise assisted in a motorized upper limb cycle ergometer (MOTOMED Viva2 REck, Reck, Baden-Wurttemberg, Germany) and a 6-channel functional electrical stimulator (TRAINFES 6 channels, TRAINFES SPA, Las condes, Chile) (see Figure 1a). The FES was installed in the wrist, elbow, and shoulder flexors and extensors using hydrogel electrodes (see Figure 1b). The activation pattern of the FES was coordinated with the cycle ergometer employing the TRAINFES inertial sensor installed on the axis of rotation of the bicycle. The configuration was made in the developer’s application in cycle ergometer mode. The intensity of the current of each channel was configured until obtaining visible muscle contraction.

**Figure 2:**
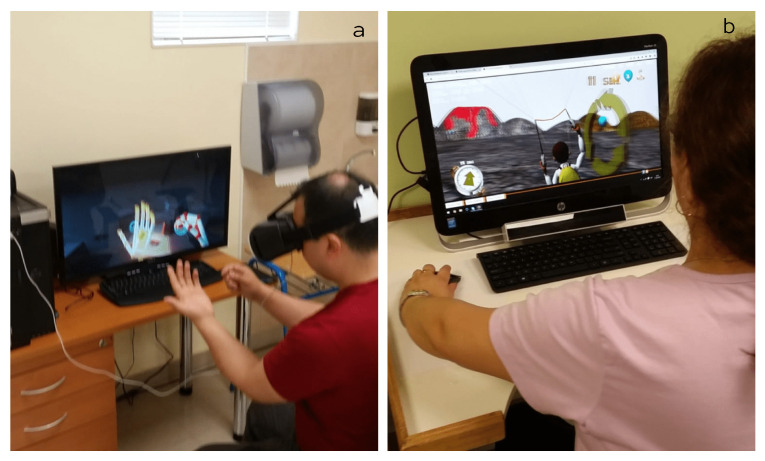
(**a**) Virtual Reality Therapy, and (**b**) Training with mixed games.

VR therapy was performed using an infrared forearm and hand movement sensor (Leap Motion, Ultraleap, San Francisco, CA, USA) and a virtual reality headset (HTC VIVE, HTC, Shenzshen, China) (see Figure 2a). Activities were carried out to promote the paretic upper limb movement in the first-person mode and on the desktop. The The training consisted of a program with mixed games(see Figure 2b). The movements trained were: flexion, extension, adduction, abduction, internal and external rotation of the shoulder, flexion and extension of the elbow, pronation of the forearm, and flexion and extension of the wrist. The virtual reality application used was the VR for rehabilitation program developed by the University of Magallanes. A kinesiologist carried out the equipment’s installation, configuration, and positioning with experience in human–computer interfaces for rehabilitation.

### 2.3. Movement Analysis

Nexus software (Oxford Metrics, Oxford, UK) was used to track the trial data, and Polygon software (Oxford Metrics, Oxford, UK) provided the kinematic outcomes of each user. In this sense, the kinematic parameters such as maximum angles reached per joint, range of motion during the task per joint, time of execution of the task, and maximum angular velocity per joint of each limb were calculated. It is essencial to mention that the movement was recorded at a sampling frequency of 100 Hz. This protocol included two modes (i.e., baseline and post-rehabilitation) to analyze the effects of the virtual reality/FES rehabilitation program. For both modes, participants were instrumented with 19 markers under a full body Plug-in Gait marker model (https://www.researchgate.net/publication/319981246_The_effects_of_the_use_of_Eye_Movement_Modelling_Examples_EMMEs_on_perceptual_and_motor_learning_RM_Human_Movement_Sciences_research_report, accessed on 21 February 2022). In addition, trials were executed on a chair and with a table in front of the patient, where ten cameras, VICON (Oxford Metrics, Oxford, UK), were distributed to acquire the user kinematics.

**Figure 3:**
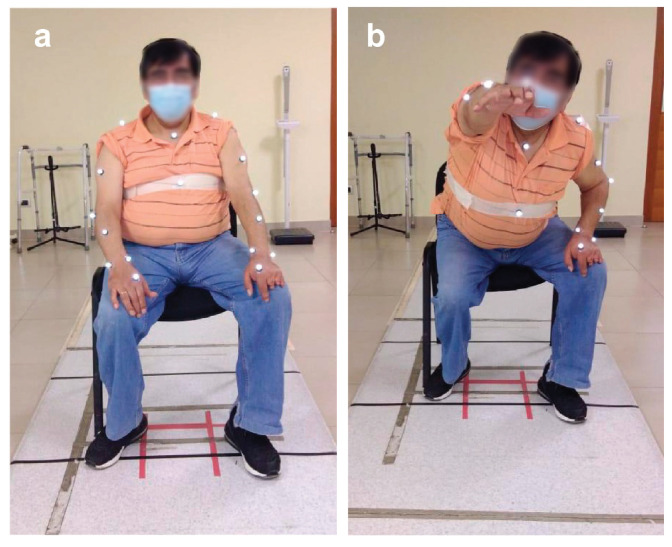
Maximum Forward Reach Test sequence. (**a**) Initial position and (**b**) Final position.

Patients performed the Maximum Forward Reach Test and the Apley Scratching Test. The first is the horizontal distance measured from the plane passing through the occipital, the scapulae, and the glutes to the vertical axis that occurs in the hand with the fingers extended forward. The distance is measured to the tip of the fingers, and the extended arm should make a 90 angle, as can be seen in Figure 3.

**Figure 4:**
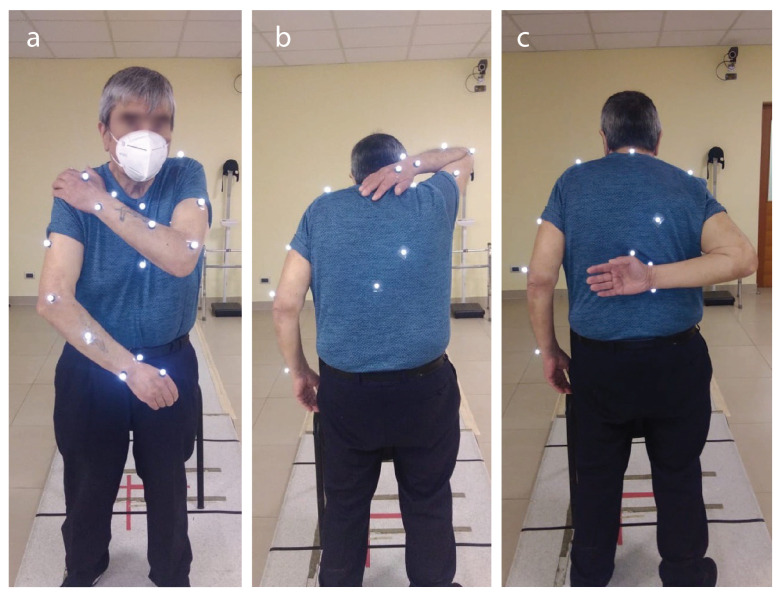
Apley Scratching Test sequence. (**a**) Action 1, (**b**) Action 2, and (**c**) Action 3.

The Apley Scratching Test consists of 3 main actions [38]:

- Action 1: The subject is instructed to touch the opposite shoulder with his hand. Here, the gloenohumeral abduction, internal rotation, horizontal abduction, and escape protraction are checked, as shown in Figure 4a.
- Action 2: The subject is told to raise his arm above his head and then bend his elbow and turn his arm out until it reaches behind his head with his palm to play with the medial edge of the counter lateral scapula or reach the column, that is, by touching the vertebrae. Here, the shoulder flexion, external rotation, and exhaust abduction are checked, as shown in Figure 4b.
- Action 3: The subject is told to reach an arm behind his back and then bend his elbow and turn his arm in with his palm out to touch the lower angle of the contralateral scapula or reach the column, that is, touch the vertebrae as far as possible. Here, the shoulder extension, internal rotation, and escape adjection are checked in Figure 4c.

### 2.4. Data Analysis

For the upper limb joints, the Wilcoxon test was used with a significance level of *p* = 0.05 to compare (i) variables of the paretic limb before and after the rehabilitation therapy and (ii) paretic non-paretic extremities. This test is used after finding a no normal distribution with a Shapiro–Wilk test.

### 2.5. Definition of Variables

To evaluate the results of this study, the clinical parameters of interest are maximum angles, RoM, execution time, and angular velocity, which are calculated with the MATLAB software.

- Maximum Angles: Its unit is in degrees, and it measures the maximum angle reached by each upper limb joint: shoulder, elbow, wrist, and forearm.
- Range of Motion: Its unit is in degrees, and it measures the rotation about a joint. The measurement of RoM is a valuable part of clinical assessment; therefore, it is essential that it is completed in a way that provides accurate and reliable results [39,40].
- Execution Time: Its unit is in seconds (s). It is the duration required to complete the exercise.
- Angular Velocity: Its unit is in (rad/s). It is derived from position or angle data.

## 3. Results

The results are presented for both activities: the Maximum Forward Reach Test and the Apley Scratching Test. The maximum angles, range of motion, angular velocities, and time of execution were analyzed.

### 3.1. Maximum Forward Reach Test

Table 2 presents the maximum angles obtained for the paretic limb in the Maximum Forward Reach Test before and after the rehabilitation therapy (RT). As shown, a significant difference was found in the flexion, extension, adduction, and int. rotation of the shoulder joint. This corresponds to the increase of the maximum angle after the rehabilitation therapy compared to before. The increase found was 16.25% for the flexion, 27.65% for the extension, 17.45% for the abduction, and 63.50% for the internal rotation of the shoulder.

Comparing the maximum angles reached between the paretic and non-paretic limb, some significant differences were found. There was an asymmetry between the paretic and non-paretic limb in three maximum angles that was not shown after the rehabilitation. In shoulder flexion, the non-paretic limb had a maximum angle 21.39% higher than the paretic limb. In shoulder adduction, the difference before was 30.98%, and in the elbow extension, the difference before was 19.37%. These results show the potential of the rehabilitation to decrease the hemiparesis presented after the stroke. These differences are observed in Table 3.

In the analysis of the RoM presented in Table 4, a significant difference was found in the shoulders’ flexion–extension and internal and external rotation. This corresponds to the increase of the RoM after the therapy compared to before. The increase found was 17.98% for flexion–extension and 18.12% for int-ext rot of the shoulder.

Comparing the RoM reached between the paretic and non-paretic limb, some significant differences were found. There was an asymmetry between the paretic and non-paretic limb in three RoM that was not shown after the rehabilitation. In the Table 5, a significant difference was found in the shoulders’ flexion–extension; the non-paretic limb had an RoM 49.39% higher than the paretic limb. In shoulder adduction–abduction, the non-paretic limb had an RoM 19.05% higher than the paretic limb. In the elbow presented in Table 5, a significant difference was found in the flexion–extension; the paretic limb had an RoM of 8.79% higher than the non-paretic limb.

Regarding the execution time, there was no significant difference before or after the rehabilitation therapy for the paretic limb, as can be seen in Table 6. For comparison of the paretic and non-paretic limb no difference was found either. However, a decrease in the paretic limb is observed after rehabilitation therapy compared to before therapy. Therefore, RoM changes do not affect execution time.

In the angular velocities, significant differences corresponded only to shoulder adduction–abduction and int–ext rotation, given an increase in angular velocities of the paretic limb after the therapy compared to before. The increase found was 39.61% for adduction–abduction and 49.01% for rotation (see Table 7).

Comparing the angular velocities reached between the paretic and non-paretic limb, some significant differences were found. An asymmetry between the paretic and non-paretic limb in four angular velocities that was not shown after the rehabilitation. In shoulder adduction–abduction, the non-paretic limb had a maximum angle 62.42% higher than the paretic limb. In the internal and external rotation of the shoulder, the difference before was 48.13%. In the wrist, a significant difference was found in the shoulders’ flexion–extension, the difference before was 73.85%. In the forearm pronation-supination, the difference before was 69.01%. Also, a significant difference was found in the shoulders’ flexion–extension movement of the shoulder and elbow; a significant difference was found before and after therapy. Before therapy, the non-paretic limb had an angular velocity 60.32% higher than the paretic limb for the shoulder and 82.70% for the elbow. After therapy, the non-paretic limb had an angular velocity 38.97% higher than the paretic limb for the shoulder and 62.40% for the elbow, which is less than before therapy (see Table 8).

### 3.2. Apley Scratching Test

Table 9 presents the maximum angles obtained for the paretic limb in the Apley Scratching Test before and after the rehabilitation therapy. As shown, a significant difference was found in the abduction of the shoulder joint. This corresponds to the increase in the maximum angle after the rehabilitation therapy compared to before. The increase found was 85.32% for the abduction.

Comparing the maximum angles reached between the paretic and non-paretic limb, some significant differences were found. There was an asymmetry between the paretic and non-paretic limb in three maximum angles that was not shown after the rehabilitation. In shoulder adduction, the non-paretic limb had a maximum angle of 22.03% higher than the paretic limb. In the abduction of the shoulder, the difference before was 37.79%. In the ext. rotation of the shoulder, the difference before was 47.97%. These results show the potential of the rehabilitation to decrease the hemiparesis presented after the stroke (see Table 10).

Analyzing the RoM presented in Table 11, a significant difference was only found in the shoulders’ adduction–abduction. RoM increases 20.23% after therapy. Comparing the RoM reached between the paretic and non-paretic limb, some significant differences were found. There was an asymmetry between the paretic and non-paretic limb in one RoM that was not shown after the rehabilitation. In shoulder adduction–abduction, the non-paretic limb had an RoM of 27.58% (see Table 12).

Concerning the execution time, a significant difference was found only before the rehabilitation therapy when comparing the paretic and non-paretic limbs. Other matches did not present any difference, as shown in Table 13. However, a decrease in the paretic limb is observed after rehabilitation therapy compared to before therapy. Therefore, RoM changes affect execution time.

Finally, in the angular velocities, significant differences corresponded only to shoulder adduction–abduction due to an increase in the angular velocities of the paretic limb therapy after therapy compared to before. The increase found was 34.65% (see Table 14).

Comparing the angular velocities reached between the paretic and non-paretic limb, some significant differences were found. There was an asymmetry between the paretic and non-paretic limb in two angular velocities, which was not shown after the rehabilitation. In the int-ext rot of the shoulder, the non-paretic limb had an angular velocity that was 72.39% higher than the paretic limb, and in the forearm pronation–supination, the difference before was 10.28%. These results show the potential of the rehabilitation to decrease the hemiparesis presented after the stroke (see Table 15).

## 4. Discussion

### 4.1. Maximum Forward Reach Test

The first important fact to consider is that the movements in which maximum angles, RoM, and angular velocities exhibited significant differences when comparing the paretic limb before and after the therapy corresponded to an increase of those variables after the therapy. As presented in Table 2, Table 4 and Table 7, values after the therapy were always higher than before the therapy when a *p*-value lower than 0.05 was obtained in the Wilcoxon test.

Another particular event to observe is that except for two movements in Table 2, all significant differences were found at the shoulder level. The differences in shoulders’ rotation and flexion/extension were presented in both RoM, while the differences in the angular velocities and shoulders’ adduction only regarded maximum angles. The results follow Cuesta-Goméz et al., who found the shoulder RoM presented in Table 4; a significant difference was found in the shoulders’ flexion–extension increase during a reaching movement [32].

Both findings are congruent also with the movement executed in the test. As described in Section 2, the Maximum Forward Reach Test includes a movement mainly of the shoulder joint. Hence, the fact that significant differences were found in movement that corresponded to the shoulder was expected. Additionally, even if no significant difference was obtained at the elbow level, mean values of the angles and RoM are lower after the therapy than before. This could be understood as an improvement by the therapy. Since muscle weakness limits the mobility of the paretic upper limb, patients with hemiparesis usually flex the elbow to shorten the lever arm and facilitates lifting in full-reach tasks [32]. The fact that elbow flexion decreases could be interpreted as a sign of minor muscle weakness.

Comparing the paretic and non-paretic limb, a major trend is clear when observing Table 3, Table 5 and Table 8: the number of variables with differences found before the therapy is diminished after the therapy. This could be an indication of the effectiveness of the therapy. More minor differences mean that both limbs are behaving more similarly, and consequently, the paretic limb is improving and getting back some movement characteristics of the non-paretic limb.

### 4.2. Apley Scratching Test

The behavior seen in the Apley Scratching Test is preresented in Table 9, Table 11 and Table 14. The values after the therapy were higher than before the therapy when a *p*-value lower than 0.05 was obtained in the Wilcoxon test.

In this case, all significant differences were found at the shoulder, this time mainly in the shoulders’ adduction/abduction regarding maximum angles, RoM, and angular velocities. Results are consistent with what was presented by Gillen et al. and Molina et al., where the abduction of the shoulder for people with hemiparesis is higher during a reaching movement [34,41].

In this case, according to the nature of the test presented in Section 2, differences could be expected at the elbow and shoulder joint level. However, differences were only found in movements that corresponded to the shoulder. This is possibly due to the characteristics of the movement performed. Since most of the arm’s weight is lifted or moved by the shoulder, and in this case, the elbow is guided by the shoulder, only changes in this joint were observed.

Concerning comparing the paretic and non-paretic limb, the trend is maintained in Table 10, Table 12 and Table 15. The number of variables with differences found before the therapy diminished after the therapy, at the point that no difference was found in the maximum angles, RoM, and angular velocities. Again, this is a display of the effectiveness of the therapy.

The new finding presented by this test is the difference found in the execution time of Table 13. The fact that the difference was seen before the therapy and not after it matches the exposed behavior. No difference in execution reflects an improvement not only considering the patient’s biomechanical but more functional abilities, which could impact his day-to-day activities.

Additionally, these methods of kinematic motion capture analysis can be incorporated into clinical practice as a gold standard for kinematic motion analysis and are increasingly implemented as an outcome measure to assess performance and quality of movement following injury or disease involving upper extremity movements [42]. Optoelectronic motion capture systems use multiple high-speed cameras that send infrared light signals to capture reflections from passive markers placed on the body. These capture systems have high accuracy and flexibility in measuring various tasks [42].

The limitations of this study are that it did not have a conventional therapy group to compare with this non-conventional rehabilitation therapy: FES and VR. In this way, the efficacy of this therapy can be determined.

## 5. Conclusions

FES has been shown to improve motor skills reacquisition in upper limbs through the performance of repetitive movements [32,43]. However, rehabilitation only with FES has proven to be insufficient for correctly performing movements toward a rehabilitation process [44]. In this study, the implementation of FES and virtual reality as complementary tools in post-stroke rehabilitation therapies has been shown to improve the range of motion, maximum angles, and angular velocities of hemiparetic upper limbs when performing two well-known motion tests (Maximum Forward Reach Test and Apley Scratching Test). The results showed that the number of variables with differences found before the therapy diminishes after the therapy. As more minor differences are observed, the paretic limb gets back movement characteristics of the non-paretic limb and performed more similarly, which is a first step in assessing the effectiveness of the therapy. When comparing the parameters of the healthy side with it before and after the training course, you can also find some changes. Their value will depend on how long patients are from the moment of stroke. FES and virtual reality have proven their benefits in the rehabilitation of post-stroke patients, as it has improved joint range of motion and maximum angular velocity.

It is important to stress that there is limited information on joint velocity analysis for post-stroke patients. Specific articles have examined reaching tasks in stroke subjects, but few include analysis of upper extremity movement [10]. Additionally, the authors found no articles related to movement analysis in post-stroke patients performing the Apley Scratching Test. Therefore, this study presents a baseline study that shows promising results regarding the functional and biomechanical improvement of post-stroke patients after technology-based rehabilitation therapy.

Future work will focus on carrying out motion analysis of activities of daily life as pouring a glass of water, opening and closing a bottle, building a bucket, and taking cutlery before and after rehabilitation therapy. In this sense, a more functional diagnostic of the improvement presented with FES and VR is expected. In addition, a comparison group receiving only conventional therapy is needed in order to improve the comparison and conclude how effective this type of non-conventional rehabilitation (FES and VR).

## Figures and Tables

**Table 1:** Clinical and demographic characteristics of patients.

Characteristics	Patients (n = 13)	%
Sex	Female 4	30.76
	Male 9	69.24
Age	Range 40–70	
Risk of facts	HTA 5	38.46
	Hypothyroidism 1	7.69
	Dyslipemia 1	7.69
	Sertraline 1	7.69
	DM 1	7.69
	Chronic dysphonia 1	7.69
	Dysarthia 1	7.69

**Table 2:** Maximum angles obtained for the paretic limb in the Maximum Forward Reach Test before and after the rehabilitation therapy.

Joint	Movement	Maximum Angle before RT (${}^{{}^{\circ}}$)	Maximum Angle after RT (${}^{{}^{\circ}}$)	*p*-Value
Shoulder	Flexion	52.17 ± 14.01	60.65 ± 12.79	*p* ≤ 0.05
	Extension	9.98 ± 6.48	12.68 ± 7.31	*p* ≤ 0.05
	Adduction	94.16 ± 46.67	110.60 ± 33.48	*p* ≤ 0.05
	Abduction	22.69 ± 20.07	16.58 ± 8.01	0.33
	Int. Rotation	29.59 ± 53.78	48.38 ± 8.73	*p* ≤ 0.05
	Ext. Rotation	63.12 ± 57.59	61.14 ± 46.61	0.08
Elbow	Flexion	84.69 ± 19.39	79.85 ± 13.38	0.54
	Extension	39.44 ± 15.91	38.02 ± 20.29	0.16
Wrist	Flexion	44.33 ± 18.97	39.14 ± 11.58	0.37
	Extension	13.44 ± 11.13	12.37 ± 10.06	0.89
Forearm	Pronation	143.51 ± 21.11	146.67 ± 18.25	0.73
	Supination	79.19 ± 88.11	110.98 ± 14.97	0.12

**Table 3:** Maximum angles Wilcoxon test of the paretic and non-paretic limb in the Maximum Forward Reach Test before and after the rehabilitation therapy.

Joint	Movement	*p*-Value before RT	*p*-Value after RT
Shoulder	Flexion	*p* ≤ 0.05	0.94
	Extension	0.13	0.41
	Adduction	*p* ≤ 0.05	0.19
	Abduction	0.29	0.23
	Int. Rotation	0.33	0.08
	Ext. Rotation	0.55	0.41
Elbow	Flexion	0.90	0.30
	Extension	*p* ≤ 0.05	0.27
Wrist	Flexion	0.90	0.27
	Extension	0.96	0.76
Forearm	Pronation	0.63	0.41
	Supination	0.56	0.42

**Table 4:** RoM obtained for the paretic limb in the Maximum Forward Reach Test before and after the rehabilitation therapy.

Joint	Movement	RoM before RT (${}^{{}^{\circ}}$)	RoM after RT (${}^{{}^{\circ}}$)	*p*-Value
Shoulder	Flex–Ext	62.15 ± 15.33	73.33 ± 14.86	*p* ≤ 0.05
	Add–Abd	116.85 ± 27.18	127.18 ± 34.45	0.06
	Int–Ext Rot	92.72 ± 33.84	109.53 ± 50.87	*p* ≤ 0.05
Elbow	Flex–Ext	124.13 ± 29.74	117.88 ± 32.66	0.37
Wrist	Flex–Ext	57.77 ± 27.04	51.52 ± 17.83	0.53
Forearm	Pron–Sup	254.97 ± 48.49	257.65 ± 29.54	0.54

**Table 5:** RoM Wilcoxon test of the paretic and non-paretic limb in the Maximum Forward Reach Test before and after the rehabilitation therapy.

Joint	Variable	*p*-Value before RT	*p*-Value after RT
Shoulder	Flex–Ext	*p* ≤ 0.05	0.73
	Add–Abd	*p* ≤ 0.05	0.56
	Int–Ext Rot	0.19	0.63
Elbow	Flex–Ext	*p* ≤ 0.05	0.83
Wrist	Flex–Ext	0.73	0.54
Forearm	Pron–Sup	0.68	0.63

**Table 6:** Execution time of the Maximum Forward Reach Test before and after the rehabilitation therapy.

Limb	Execution Time before RT (s)	Execution Time after RT (s)	*p*-Value
Paretic	23.57 ± 13.00	8.10 ± 2.46	0.27
Non-paretic	8.49 ± 2.64	8.85 ± 4.18	0.42
*p*-Value	0.12	0.17	

**Table 7:** Angular velocities obtained for the paretic limb in the Maximum Forward Reach Test before and after the rehabilitation therapy.

Joint	Movement	Angular Velocity before RT (rad/s)	Angular Velocity after RT (rad/s)	*p*-Value
Shoulder	Flex–Ext	148.95 ± 60.14	165.77 ± 71.86	0.67
	Add–Abd	232.80 ± 128.11	325.03 ± 197.75	*p* ≤ 0.05
	Int–Ext Rot	245.81 ± 111.54	366.29 ± 205.36	*p* ≤ 0.05
Elbow	Flex–Ext	136.56 ± 51.50	176.55 ± 123.12	0.54
Wrist	Flex–Ext	198.11 ± 90.30	181.32 ± 88.87	0.63
Forearm	Pron–Sup	220.05 ± 96.41	207.63 ± 90.14	0.58

**Table 8:** Angular velocity Wilcoxon test of the paretic and non-paretic limb in the Maximum Forward Reach Test before and after the rehabilitation therapy.

Joint	Variable	*p*-Value before RT	*p*-Value after RT
Shoulder	Flex–Ext	*p* ≤ 0.05	*p* ≤ 0.05
	Add–Abd	*p* ≤ 0.05	0.27
	Int–Ext Rot	*p* ≤ 0.05	0.33
Elbow	Flex–Ext	*p* ≤ 0.05	*p* ≤ 0.05
Wrist	Flex–Ext	*p* ≤ 0.05	0.19
Forearm	Pron–Sup	*p* ≤ 0.05	0.14

**Table 9:** Maximum angles obtained for the paretic limb in the Apley Scratching Test before and after the rehabilitation therapy.

Joint	Movement	Maximum Angle before RT (${}^{{}^{\circ}}$)	Maximum Angle after RT (${}^{{}^{\circ}}$)	*p*-Value
Shoulder	Flexion	62.88 ± 9.64	67.21 ± 12.04	0.24
	Extension	42.76 ± 12.16	34.41 ± 20.39	0.10
	Adduction	98.91 ± 21.02	104.69 ± 29.19	0.10
	Abduction	16.35 ± 11.37	30.30 ± 28.75	*p* ≤ 0.05
	Int. Rotation	102.36 ± 15.65	110.66 ± 22.60	0.21
	Ext. Rotation	54.42 ± 28.64	62.31 ± 37.27	0.20
Elbow	Flexion	144.01 ± 7.77	143.40 ± 9.04	0.37
	Extension	61.63 ± 18.18	67.51 ± 15.27	0.19
Wrist	Flexion	49.70 ± 18.31	52.12 ± 16.24	0.78
	Extension	21.06 ± 13.65	15.94 ± 15.27	0.19
Forearm	Pronation	147.65 ± 15.39	148.12 ± 11.64	0.94
	Supination	57.18 ± 32.39	58.99 ± 27.15	0.78

**Table 10:** Maximum angles Wilcoxon test of the paretic and non-paretic limb in the Apley Scratching Test before and after the rehabilitation therapy.

Joint	Variable	*p*-Value before RT	*p*-Value after RT
Shoulder	Flexion	0.54	0.94
	Extension	0.58	0.78
	Adduction	*p* ≤ 0.05	0.83
	Abduction	*p* ≤ 0.05	0.30
	Int. Rotation	0.16	0.94
	Ext. Rotation	*p* ≤ 0.05	0.63
Elbow	Flexion	0.24	0.27
	Extension	0.83	0.58
Wrist	Flexion	0.63	0.58
	Extension	0.33	0.63
Forearm	Pronation	0.89	0.63
	Supination	0.94	0.58

**Table 11:** RoM obtained for the paretic limb in the Apley Scratching Test before and after the rehabilitation therapy.

Joint	Movement	RoM before RT (${}^{{}^{\circ}}$)	RoM after RT (${}^{{}^{\circ}}$)	*p*-Value
Shoulder	Flex–Ext	105.74 ± 15.26	101.63 ± 28.54	0.78
	Add–Abd	112.27 ± 29.37	134.99 ± 47.28	*p* ≤ 0.05
	Int–Ext Rot	156.78 ± 39.14	172.98 ± 43.44	0.08
Elbow	Flex–Ext	205.64 ± 19.23	210.91 ± 17.00	0.33
Wrist	Flex–Ext	70.77 ± 22.24	68.06 ± 22.41	0.54
Forearm	Pron–Sup	204.83 ± 35.26	207.12 ± 30.09	0.90

**Table 12:** RoM Wilcoxon test of the paretic and non-paretic limb in the Apley Scratching Test before and after the rehabilitation therapy.

Joint	Variable	*p*-Value before RT	*p*-Value after RT
Shoulder	Flex–Ext	0.12	0.78
	Add–Abd	*p* ≤ 0.05	0.63
	Int-Ext Rot	0.06	0.78
Elbow	Flex–Ext	0.58	0.09
Wrist	Flex–Ext	0.49	0.41
Forearm	Pron–Sup	0.94	0.89

**Table 13:** Execution time of the Apley Scratching Test before and after the rehabilitation therapy.

Limb	Execution Time before RT (s)	Execution Time after RT (s)	*p*-Value
Paretic	19.59 ± 8.06	16.64 ± 11.12	0.12
Non-paretic	13.58 ± 5.72	14.29 ± 3.56	0.37
*p*-Value	*p* ≤ 0.05	0.83	

**Table 14:** Angular velocities obtained for the paretic limb in the Apley Scratching Test before and after the rehabilitation therapy.

Joint	Movement	Angular Velocity before RT (rad/s)	Angular Velocity after RT (rad/s)	*p*-Value
Shoulder	Flex–Ext	263.33 ± 90.59	311.73 ± 132.98	0.21
	Add–Abd	338.89 ± 178.46	456.33 ± 231.76	*p* ≤ 0.05
	Int-Ext Rot	429.35 ± 220.98	571.96 ± 278.76	0.06
Elbow	Flex–Ext	296.10 ± 135.79	314.97 ± 149.73	0.37
Wrist	Flex–Ext	376.43 ± 153.98	410.92 ± 186.49	0.41
Forearm	Pron–Sup	471.67 ± 208.93	498.33 ± 228.28	0.68

**Table 15:** Angular velocity Wilcoxon test of the paretic and non-paretic limb in the Apley Scratching Test before and after the rehabilitation therapy.

Joint	Variable	*p*-Value before RT	*p*-Value after RT
Shoulder	Flex–Ext	0.73	0.9
	Add–Abd	0.08	0.73
	Int-Ext Rot	*p* ≤ 0.05	0.65
Elbow	Flex–Ext	0.10	0.08
Wrist	Flex–Ext	0.68	0.78
Forearm	Pron–Sup	*p* ≤ 0.05	0.63

## Data Availability

Not applicable.
